# S04 Making Sense of Physical Activity Policy Assessment: Lessons Learned, Challenges, Next Steps

**DOI:** 10.1093/eurpub/ckae114.210

**Published:** 2024-09-26

**Authors:** Peter Gelius, Petru Sandu

**Affiliations:** Institute of Sport Sciences, Université de Lausanne; University Babes-Bolyai, Cluj-Napoca

## Abstract

Increasing population levels of physical activity is an important global health priority. The World Health Organization and many countries have developed evidence-based public health recommendations and action plans to do so. However, implementing these recommendations and plans remains limited in almost all countries, even Europe, where priorities and resources seem well aligned. Several research groups have developed policy assessment tools to understand better these challenges to evaluate policy development and implementation. The process has proved to be complex for several reasons. Increasing physical activity among populations depends upon actions in many sectors. Thus, policies must engage and include sectors beyond health. While policy is typically developed nationally, implementation often occurs at sub-national levels such as states and cities. Balancing the need for collecting complex data across multiple sectors and government levels while keeping instruments short enough to be useful for public health practitioners and appealing to policymakers takes work. In this session, we will hear from six research groups that have developed and applied policy assessment tools in Europe, Latin America, and Japan at national and sub-national levels. Each presenter will share their experiences, strengths, and weaknesses of the instruments, challenges, and planned next steps. The brief presentations will be followed by a panel discussion guided by a discussant to synthesize the research to date, summarize progress and remaining challenges, and suggest the way forward for both policy research and public health practice.

